# Exploring the oral microbiome: an updated multidisciplinary oral healthcare perspective

**DOI:** 10.15190/d.2023.4

**Published:** 2023-06-30

**Authors:** Aman Chowdhry, Priyanka Kapoor, Deepak Bhargava, Dinesh Kumar Bagga

**Affiliations:** ^1^School of Dental Sciences, Sharda University, Greater Noida (UP), India; ^2^Department of Oral Pathology & Microbiology, Faculty of Dentistry, Jamia Millia Islamia, New Delhi, India; ^3^Department of Orthodontics, Faculty of Dentistry, Jamia Millia Islamia, New Delhi, India; ^4^Department of Oral Pathology and Microbiology, School of Dental Sciences, Sharda University, Greater Noida (UP), India; ^5^Department of Orthodontics, School of Dental Sciences, Sharda University, Greater Noida (UP), India

**Keywords:** Biofilm, dysbiosis, microbiome, 16S rRNA, microorganism, genus, microflora, HOMD.

## Abstract

The oral cavity is home to diverse microbial content, collectively called as the oral microbiome. The latest technological advancements have unraveled the intricacies of the oral microbiome.  It can be of great importance for oral health care givers to know the fundamentals and latest developments in the field of the oral microbiome, as oral dysbiosis is associated with many common diseases frequently seen and managed by them. These diseases include dental caries, periodontitis, mucosal diseases (such as oral leukoplakia, oral lichen planus, and systemic lupus erythematosus), oral cancers, and even co-infections related to the current COVID-19 pandemic. The emergence of new genomic and molecular biology methodologies has been pivotal for understanding the role of the human microbiome in health and disease. The current review compiles oral microbiome in health and disease with a multidisciplinary dental approach. The insight into the oral microbiome, which is provided dental specialty wise in the current article will initiate and guide researchers of various disciplines in developing microbiome-based therapeutic or prophylactic management strategies, managing public health challenges by microbiome-based boarder interventions and divert resources for preserving and achieving a balanced oral microbiome.

## 1. Introduction

Microbiome is a term coined by Joshua Lederberg and is used to describe human body resident microorganisms^1^. Together with resident micro-organisms humans have co-evolved and co-existed for millions of years. These microorganisms existing in the human body can be pathogenic (i.e. disease causing) or commensal (i.e. normal residents) or symbiotic (i.e. interdependence). The symbiotic microbial residents along with the host humans form a 'superorganism', and are also known as "holobiont"^1,2^. Kilian M et al 2016 have enumerated the positive effects of host-microbiome symbiosis. Some of these positive effects include "providing additional metabolic potential", "regulation of cardiovascular system", "resistance to colonization by pathogens", "maintenance of healthy digestive tract" and "supporting host defence"^2^.

The digestive tract of humans consists of the oral cavity, esophagus, stomach, small intestine, large intestine, and colon. The composition of the microbiome varies in each part of the track and is even more unique in the case of the oral cavity due to the presence of teeth and hard tissue^3-5^. Oral cavity microbiota has most abundant counts of Firmicutes, whereas the stool microbiota is dominated by Bacteroidetes^4^.

**Figure 1:**
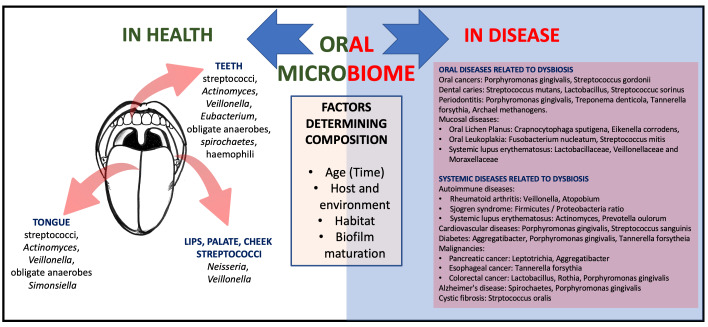
Factors determining composition of oral microbiome and catalogued oral microbiome groups in health and disease. Adapted, modified and compiled from references^7-13^

Collectively, microorganisms found in the human oral cavity or "oral microbiome" are also referred to as oral microbiota or oral microflora^6^. Factors determining composition of oral microbiome^7^ and catalogued oral microbiome groups in health^8^ and disease^9-13^, presented in Figure 1. The insight of the oral microbiome provided in the current article may help various health care researchers and practitioner’s (including dentists) world over to develop multidisciplinary strategies for preserving a balanced oral microbiome. These strategies become even more important and relevant in the current pandemic COVID-19 context.

## 2. Oral microbiome in human health

The oral cavity is home to the second most diverse microbial content in the body after gut and harbors around 700 taxa of bacteria^1^. Dutchman Antony van Leeuwenhoek who is considered as "father of microbiology" is credited for being the first to identify the oral microbiome. He examined his dental plaque and called the microbes "Dierken", which translates to small lively objects^1,2,14^.

Mostly the mouth of a newborn is sterile, and inoculation of microorganisms starts from the first feed onwards. The first microbes to colonize are called “pioneer species”, which collectively form the “pioneer microbial community”. Microbial succession leads to a stable situation with diverse resident oral microflora^8^. In adult human life the oral microbiome composition is in a balanced stable state (called "microbial homeostasis"), which comprises both natural and repeated colonizers of mouth without any evident consequences on oral health^15^. Kaan AMM et al 2021 have discussed the acquisition and establishment of normal oral microbial flora during various phases of human life^16^. The term “Oralome” has been proposed to define interactions between host and oral microbiome^17^. Some of the bacteria in healthy oral cavity consists are gram-positive rod (Actinomyces, Bifidobacterium), gram-positive cocci (Abiotrophia, Peptostreptococcus), gram-negative rods (Campylobacter, Capnocytophaga), and gram-negative cocci (Moraxella, Neisseria)^1^.

The "human microbiome project" launched by the National Institute of Health is a summation of multiple projects that recognizes the importance of exploring the human microbiome^18,19^. Human Oral Microbiome Database (HOMD) launched by the National Institute of Dental and Craniofacial Research (NIDCR) is a repository of oral microbiome with in-depth descriptions about them for application in the scientific community. The HOMD provides scientist with a “body site-specific” comprehensive database of taxonomic and genomic information^20^. Out of 774 oral bacterial species present, “58% are officially named, 16% unnamed but cultivated and 26% are known only as uncultivated phylotypes”^21^.

## 3. Oral microbiome in disease

The oral cavity serves as a valuable interaction site for exchange of internal environment and the exogenous substances and hence the constituents of oral microbiome undergo continuous transformation. The oral microbiome can be friends and foe too, i.e. oral microbiome interaction with the human body can be protective and pathogenic^22^. Disruption of normal microflora is known as dysbiosis^7,22^. Factors contributing to dysbiosis include saliva, oral hygiene, gingivitis, diet, and smoking^2^. Oral dysbiosis can contribute to various oral diseases such as dental caries^23-25^, periodontitis^25-27^ and even oral cancers^28^. Oral microbiome is also closely related to systemic diseases^29^ as well. These systemic diseases include adverse pregnancy outcomes^30^, rheumatoid arthritis^31^, and cardiovascular disease^32^.

Hence, ecological preventive measures that go beyond conventional preventive measures are required in a multidisciplinary environment. Important strategies which can exploit exclusive properties of the healthy oral microbiome are being developed^33^. We discuss four main oral concerns i.e. dental caries, periodontitis, mucosal diseases and oral cancers in the oral microbiome context.

### 3.1 Dental Caries

**“**Dental plaque is a structurally- and functionally-organized biofilm”^34^, which is rich in various species of microorganism^35^. The dental caries lesion on the tooth surface can be progressive or arrested, either way, it reflects in the activity of the covering biofilm^36^. The components of biofilm of tooth crown and root surface differ from each other due to effect of gingival crevicular fluid^37^. Coronal caries tooth can be free from root caries and vice versa^38^.

Early childhood caries (ECC) affects about 70% of children in some countries. The oral microbiome plays a major role in ECC and saliva testing of infants may help in the prediction of its risk^39^. Lif Holgerson P et al. ^40^ have concluded that numerous taxa of oral biofilms of 3-year-old individuals can be linked to the presence (including Actinobaculum, Aggregatibacter, Atopobium, Streptococcus genera) or even absence of dental caries.

Dental biofilm and dental caries: the biofilm has been defined by Donlan and Costerton as "a microbially derived sessile community characterized by cells that are irreversibly attached to a substratum or interface or to each other, are embedded in a matrix of extracellular polymeric substances that they have produced, and exhibit an altered phenotype with respect to growth rate and gene transcription”^41^. Dental biofilm or oral biofilm is dynamic and a metabolically functional structure. Formation oral biofilm appears to be influenced by very large changes in protein expression over time and cariogenic microorganisms. These microbes produce acids (lactic acid, formic acid, acetic acid, propionic acid, etc.), which are a by-product of carbohydrate breakdown. The presence of these acids leads to pH below 5.5, which subsequently leads to demineralization and proteolytic breakdown of hard tissues of tooth^36^.

### 3.2 Periodontitis

Inflammation of periodontium (Periodontitis) after bacterial infection is very common in humans. This inflammation is a consequence of a compromised host immune response to bacterial taxa due to dysbiosis of the oral microbiome^26,42,43^. Periodontitis and clinical parameters of periodontal destruction has been linked to increased Interleukin-17 expression, which plays as a key mediator in this immunopathology^44^.

More than 400 phenotypes have been found in periodontal pockets in periodontitis^45^. The bacteria associated with severe periodontitis (called “Red complex bacteria”) are, Treponema denticola, Tannerella forsythia, and Porphyromonas gingivalis^46^. Guerra F et al.^47^ have derived *Porphyromonas gengivalis* and *Streptococcus *(S.) *mutans* as risk factors for progression of periodontitis, and Aggregatibacter actinomycetemcomitans have a protective role related to periodontitis.

Host response may be compromised in systemic diseases such as systemic lupus erythematosus, rheumatoid arthritis, and diabetes. These diseases increase the host oral cavity susceptibility to destructive periodontal diseases^29^. Results of a study conducted by Chen C et al 2018 have revealed differences in species and assembly processes of oral microbiome between periodontium during health and disease. Their experimental assembly analysis had led to the identification of potentially effective approaches for managing periodontitis^26^. Oral Probiotics, Prebiotics, and Symbiotic can be utilized in management of dysbiosis produced in periodontists^48^.

### 3.3 Mucosal diseases

Oral cavity is lined by mucosal membrane and provides rich environment in which oral microbes can flourish^6^. It is established by many studies that microorganisms play an important role in mucosal diseases^14^. Mucocutaneous diseases including oral leukoplakia^11,49^, oral lichen planus^50,51^, and systemic lupus erythematosus^52^ are oral microbiota associated. Amer et al.^11^ and Decsi et al.^12^ have reported increased Fusobacterium nucleatum and decreased Streptococcus mitis in oral leukoplakia when compared with microbial abundance in healthy mucosa of oral cavity. Yu et al. found increased microbial groups of *Lautropia* and *Gemella *in erosive oral lichen planus and, *Haemophilus* and *Neisseria *in non-erosive oral lichen planus*. *Abiotrophia and *Oribacterium *were found to be relatively abundant in both erosive oral lichen planus and non-erosive lichen planus^13^. Li et al. study has concluded that Lactobacillaceae, Veillonellaceae and Moraxellaceae were enriched in oral microbiota of systemic lupus erythematosus^53^.

Oral microbial dysbiosis has been identified in systemic autoimmune diseases. It has been suggested “genomics, transcriptomics, proteomics and metabolomics technology” should be used to investigate the oral microbiome of systemic autoimmune diseases^54^. Microbiological aspects of common diseases of the oral mucosa (especially autoimmune mediated) is of importance for dental experts, especially the "triple O" (i.e., oral surgeons, oral pathologists, and oral medicine specialists).

### 3.4 Oral Cancers

Every year a considerably high number of new invasive cancer cases are diagnosed. Out of these cancers, oral cancer has a very high incidence^55^. Although the microbiome differs in the healthy oral cavity and oral squamous cell carcinoma (OSCC), the pathogenic bacteria or bacterial spectrum associated with OSCC is yet to be identified^28^.

Periodontitis is associated with dysbiosis and patients suffering from periodontitis have a 2-5 times more risk of any cancer^56^. A correlation between periodontal disease and many cancer types^57^ including oral cancers exists^57-60^.

Some oral microbiome taxa such as Porphyromonas gingivalis and Fusobacterium nucleatum have carcinogenic properties like inhibiting apoptosis, activating cellular multiplication, inducing chronic inflammation, promoting cellular invasion, and production of carcinogens^56^. The causal relationship between the oral microbiome and oral cancers is complex and needs to be explored. Oral microbiome-based research can be the key to early diagnosis of OSCC, and enable modulation of the oral microbiome for prophylactic and therapeutic applications^28^.

## 4. Oral microbiome in a multidisciplinary dental perspective

Preserving the oral microbiome through an interdisciplinary and multidisciplinary approach coupled with the latest technological support can lead to a positive transformation of overall oral health. All sub-disciplines of dentistry are affected by the flora of the mouth or have direct implications in patients' care. Hence practitioners from all branches of dentistry should be abreast in the fundamentals of the oral microbiome and should apply its fundamentals in daily dental practice. We attempt to update and discuss the clinical implications of dental disciplines from an oral microbiome perspective.

### 4.1 Restorative Dentistry

Endodontic infections are polymicrobial in nature^61^. Phylum belonging to *Actinobacteria*, *Firmicutes*, *Proteobacteria* and *Bacteroidetes* have been found in infected root canals^62^. Presence of increased pathogenic microbe groups and decrease/absence of commensal bacteria is often an indication of early stages of periapical disease^63^. Removing pathogenic microorganisms and stop them from further infecting or reinfecting the apical and/or periapical areas of tooth is one of the primary goals of endodontic restorations^61^. The integrity of the dental restoration is continuously challenged by the oral microbiome's metabolic actions and can lead to a "less-than-desirable half-life" of the composite restorations in usage. To counter this microbial challenge, various novel antimicrobial composites restorations are being developed^64^.

### 4.2 Oral and maxillofacial surgery

Understanding oral microbiome and its role post-surgery is of paramount importance for clinicians especially oral and maxillofacial surgeons. Clinical over-prescription of antibiotic drugs may have played an important part in progression of antibiotic resistance^655^. Usage of probiotics improves healing of oral wounds^66^ and it may be practical alternate to managing wounds of oral cavity by antibiotics.

Lee W et al.^67^ has suggested oral surgeons to carry out more research related to oral microbiome and oral and maxillofacial diseases. Liu et al.^68^ in their study, sequencing v3-v4 hypervariable regions of the 16S ribosomal RNA (16S rRNA) gene indicated that the risk of post-operative inflammation at graft location is related to oral microbiome taxa before alveolar bone grafting. Pushalkar S et al 2014 have suggested that novel bacterial species along with deficient innate immune response may be associated with the pathogenesis of osteonecrosis of jaws^69^.

### 4.3 Orthodontics

Fixed orthodontic appliance treatment due to its prolonged treatment time makes the patient prone to periodontal inflammation, caries, white spot lesions (WSLs), due to change in oral flora with a relative abundance in obligate anaerobes (including periodontal pathogens), S. mutans and Lactobacillus and a decrease in commensal bacteria^70,71^. On the other hand, aligners are set of removable appliances which have shown insignificant changes in the oral microbiome^72^. Additionally, conventional brackets in comparison with self-ligating show significantly higher S. mutans level in later part of treatment, thus increasing susceptibly to white spot lesions (WSLs) and caries^71^. But when compared to healthy individuals, the oral microbiome of orthodontic patients has shown a greater number of Pseudomonas species. The finding of dysbiosis in orthodontic patients can have a predictive value for oral complications and may form the basis for future precision targeted therapies in orthodontics^73^. Oral microbiome screening in combination with quantifying acidogenicity in saliva or dental plaque before or during orthodontic management of patient may confer positive results to the susceptible orthodontic patients and timely intervention can prevent the development of WSLs^71^, or opportunistic infections like Candida albicans^74^.

### 4.4 Periodontics

Though periodontitis has been discussed in the earlier part of our article, it's important to emphasize few important points from the periodontist perspective. Periodontal diseases start by bacteria that gather in dental biofilm at the tooth surface and subsequently affect the nearby periodontal structures^29^. Periodontitis is primarily determined by the entire microbiome rather than being associate with a single pathogen^47^. Hence, a broad spectrum instead of a narrow spectrum approach is suggested. Periodontal inflammation is associated with multiple systemic conditions including cardiovascular diseases, cancer and pediatric obstructive sleep apnea (pOSA)^75^ amongst many others. Hence, we suggest having a systemic approach to microbiome, along with meta-genomic approach since microbial and immunological triggers links may be associated with each of these conditions.

### 4.5 Prosthodontics

**Age changes**: As the host ages, the microbiome becomes increasingly complex^7^. Elderly individuals with already deteriorated general health or compromised immunity can foster many opportunistic pathogens. These opportunistic pathogenic infections include staphylococci, enterobacteria, pseudomonads, and yeasts.

**Dental Implants:** Biofilms form on implant surfaces in the oral cavity^76^. Metagenomics and metatrascriptomics based analysis of oral microbiome is increasing the knowledge and perception of the peri-implant infections. Conventional laboratory-based analysis microbiome analysis has revealed that healthy dental implants are associated with aerobic Gram-positive cocci, whereas its pathological failure is associated with Gram-negative anaerobic rods^77^.

**Dentures:** Dental plaques and denture plaques are different significantly distinct both in composition and diversity^78^. Malodourous dentures (rated organoleptically) have shown higher microbiome diversity^79^. Similarly, diverse oral microbiome ecosystem has been noted in removable partial denture (RPD) wearers on a denaturing gradient gel electrophoresis study. The health-associated genera decrease, whereas the disease-associated genera increase significantly on wearing RPDs^80^.

### 4.6 Pedodontics

The oral microbiome associated with various health conditions including Celiac diseases, autism, Henoch-schönlein purpura disease, pediatric appendicitis, pediatric inflammatory bowel disease, pediatric obstructive sleep apnea syndrome and ECC has been summarized earlier^81^. Pyrosequencing study which was based on 16S rRNA gene V1-V3 hypervariable regions revealed that the oral microbiome has association with severe ECC^82^. Animal^83^ and human^84^ based studies have shown that young children with iron deficiency are at risk of ECC. Strategies suggested for the prevention of ECC include preventing *S. mutans* transmission, carbohydrate control, brushing of teeth, early dental examinations, and fluoride applications.

### 4.7 Oral Pathology and oral medicine

Latest advances in technology have unraveled the intricacies of the oral microbiome. This progress is leading to increased evidence of oral microbiome (taxa and abundance) role in various systemic and oral health conditions^10^. Its established that few oral premalignancy are associated with a change in the oral microbiome^85-87^. Hernandez BY et al 2017 found significantly high levels of S. mutans, Actinomyces, and Oribacterium in beetle nut chewers having oral lesions^86^. Amer A et al 2017 reported that oral leukoplakia exhibits an altered oral microbiome (co-occurrence of Campylobacter, Leptotrichia, and Fusobacterium species)^11^, which was similar microbiome observed in colorectal cancer^11,88^. Association of P. *gingivalis* infection has been found in “oro-digestive cancers, increased oral cancer invasion, and proliferation of oral cancer stem cells”^57^.

The emergence of new genomic methodologies, which include bioinformatics technology and next-generation sequencing have been pivotal in understanding the role of the human microbiome in health and disease^2^. Oral pathology and Oral Medicine subjects can be torchbearers in this quest of exploring the oral microbiome, as the very nature of their subject enables them to clinic-pathologically explore the microbiome. We suggest both the subjects, along with all the other stakeholders divert resources for translational research. All this is to develop precise and effective therapeutic strategies so that oral health care (OHC) workers can utilize the protective properties of the resident flora.

### 4.8 Community Dentistry

Active maintenance of a healthy microbiome rather than management of the disease should be the primary concern for OHC givers. Fundamentals of oral flora with their clinical implications should be part of community dentistry training of both graduates and postgraduates. Grigalauskienė R et al 2015 have suggested approach dental caries as biological phenomenon during its management^89^. The trained students and dental professionals can teach patients correct lifestyle choices, plaque control techniques, and oral hygiene habits for maintaining a healthy oral microbiome. Anti-tobacco and controlled diabetes can be part of microbiome-based community propagation of OHC programs.

Currently, most of the microbiome based therapeutic or prophylactic treatments are focused on individual or clinical levels. However, managing public health challenges by microbiome-based boarder interventions has promising prospects. Adding probiotics or prebiotics for supporting or supplementing diet is one such initiative which can have mass benefits^90,91^.

## 5. Oral microbiome and COVID-19

World Health Organization declared corona virus disease of 2019 (COVID-19) as a pandemic on 11^th^ March 2020^92,93^. The characteristics of the corona virus are still unknown, and the new information which keeps adding each day due to research changes diagnostic and management approaches^94^. During this pandemic, various cases of co-infection with other viruses, bacteria, and fungi have been reported. Some of these pathogens have origin from the oral cavity^95^.

There is presence of oral dysbiosis in COVID-19 patients^96^, and elevated abundance of *Granulicatella* and *Rothia mucilaginosa* is seen in the oral microbiome of COVID-19 patients^97^. Decreased species richness and alpha-diversity value has also been reported in COVID-19 patients as compared to healthy controls^96^. Opportunistic pathogens of oral cavity (e.g. Capnocytophaga and Veillonella) have been found in the bronchoalveolar fluid of COVID-19 patients^95^. Increased abundance of genera “*Prevotella*, *Lactobacillus*, *Capnocytophaga*, *Porphyromonas*, *Abiotrophia*, *Aggregatibacter*, and *Atopobium” *seen in periodontitis, poor oral hygiene and COVID-19 patients, which suggests microbiological correlation amongst them^96^. There is an association between COVID-19 severity and periodontal symptoms^98^. Bao L et al. have recommended oral hygiene in COVID-19 patients should be a concern, since co-infection due to poor hygiene can be critical to prognosis^95^.

Relationship amid oral microbiome, inflammation, and mucosal IgA response suggest that oral microbiome profiling may help in finding susceptibility to COVID-19 infection of an individual^96^. Although the human oral microbiome plays an important role in immune homeostasis, still its association with susceptibility and severity of COVID-19 infection is not completely understood.

## 6. Conclusion

**Figure 2:**
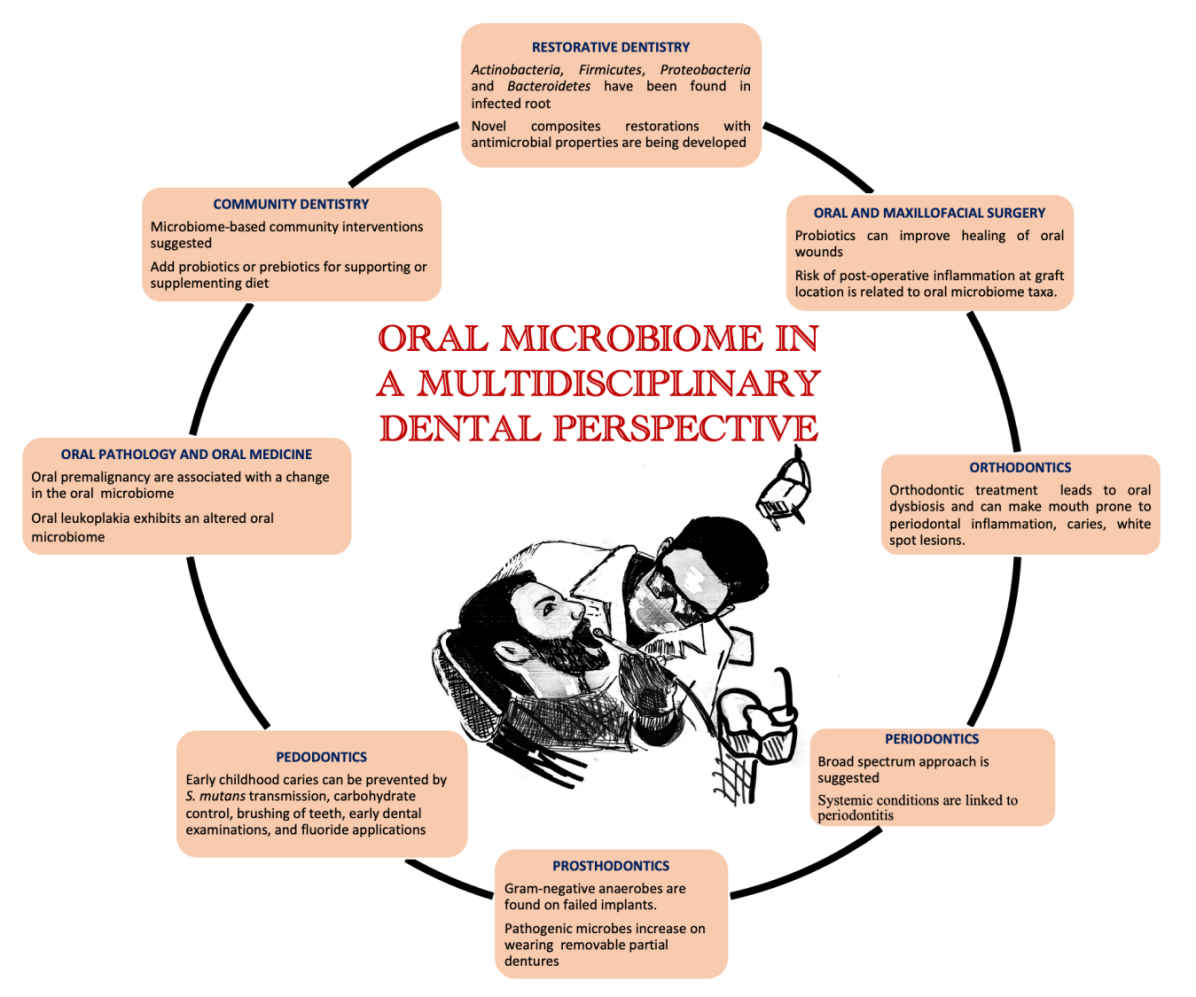
Summary and points to remember about oral microbiome in a multidisciplinary dental perspective

With advancements in science and technology, the complexities of the oral microbiome are being solved. Through this review, we have tried to explore the fundamentals of the oral microbiome in health and disease. Further, we have tried to highlight a comprehensive approach to variation in oral microbiome in different pathological conditions and the clinical relevance to multiple dental disciplines. Figure 2 summarizes oral microbiome in a multidisciplinary dental perspective.

The evaluation of oral microbiome is becoming more relevant and important for OHC givers, as oral dysbiosis is associated with dental caries, periodontitis, mucosal diseases, oral cancers, and even in co-infections related to the current pandemic COVID-19. The current article can initiate and help various health care researchers and practitioner’s world over, especially those related to OHC to develop management strategies and divert resources for preserving a balanced oral microbiome.
